# Histone methyltransferase KMT2D cooperates with MEF2A to promote the stem-like properties of oral squamous cell carcinoma

**DOI:** 10.1186/s13578-022-00785-8

**Published:** 2022-04-27

**Authors:** Xinmiao Wang, Rui Li, Luping Wu, Yang Chen, Shaopeng Liu, Hui Zhao, Yifan Wang, Lin Wang, Zhe Shao

**Affiliations:** 1 The State Key Laboratory Breeding Base of Basic Science of Stomatology (Hubei-MOST) and Key Laboratory for Oral Biomedicine of Ministry of Education (KLOBM), School and Hospital of Stomatology, Wuhan University, Wuhan, 430089 China; 2 Department of Oral and Maxillofacial-Head and Neck Oncology, School of Stomatology-Hospital of Stomatology, Wuhan University, Wuhan, China; 3 Department of Stomatology, Union Hospital, Tongji Medical College, Huazhong University of Science and Technology, Wuhan, 430022 China; 4 School of Stomatology, Tongji Medical College, Huazhong University of Science and Technology, Wuhan, 430030 China; 5 Hubei Province Key Laboratory of Oral and Maxillofacial Development and Regeneration, Wuhan, 430022 China

**Keywords:** KMT2D, MEF2A, Stemness, OSCC, CTNNB1, Organoid

## Abstract

**Background:**

Epigenetic reprogramming is involved in multiple steps of human cancer evolution and is mediated by a variety of chromatin-modifying enzymes. Specifically, the histone lysine methyltransferase KMT2D is among the most frequently mutated genes in oral squamous cell carcinoma (OSCC). However, the mechanisms by which KMT2D affects the development of OSCC remain unclear.

**Results:**

In the present study, we found that the expression of KMT2D was elevated in OSCC compared to paracancerous specimens and was correlated with a more advanced tumor grade. More importantly, knockdown of KMT2D impaired their reconstitution in patient-derived organoids and decreased the expression of CD133 and β-catenin in OSCC cells. In in vitro and in vivo models, knockdown of KMT2D reduced the colony formation, migration and invasion abilities of OSCC cells and delayed tumor growth. Mechanistically, the dual-luciferase reporter and co-immunoprecipitation assays in two individual OSCC cell lines indicated that KMT2D may cooperate with MEF2A to promote the transcription activity of CTNNB1, thereby enhancing WNT signaling.

**Conclusion:**

The upregulation of KMT2D contributes to stem-like properties in OSCC cells by sustaining the MEF2A-mediated transcriptional activity of CTNNB1.

**Supplementary Information:**

The online version contains supplementary material available at 10.1186/s13578-022-00785-8.

## Background

Head and neck cancer, including oral squamous cell carcinoma (OSCC), is the sixth-most common cancer worldwide. Due to the complexity of genetic and epigenetic variant burdens, only 40–50% of patients with OSCC survive for more than 5 years [1, 2]. A better understanding of the molecular landscape of OSCC may be beneficial for updating clinical treatments. Specifically, epigenetic reprogramming, including DNA methylation and various histone modifications, has been increasingly recognized as a novel hallmark of cancer [3]. Such activity is dynamically orchestrated by a large subset of chromatin-modifying enzymes, such as histone methyltransferase (HMT) and demethylase (HDM), which facilitate the addition or removal of methyl upon histone [3, 4]. It has been widely reported that the aberrant expression of several chromatin-modifying enzymes is closely correlated with tumor relapse, metastasis, and drug resistance, leading to the potential for targeting key epigenetic mediators in patients with cancer [3–7].

Of chromatin modifiers, histone-lysine *N*-methyltransferase 2D (KMT2D) is among the most frequently mutated genes in multiple human cancers, including OSCC [8, 9]. The Cancer Genome Atlas Network (TCGA) revealed that the mutation frequency of KMT2D in head and neck squamous cell carcinoma (HNSCC) was 18% among 279 HNSCC patients [10]. Additionally, through whole-exon sequencing of 86 OSCC tissue samples, we revealed that the mutation type of KMT2D in OSCC was mainly missense mutation [11]. Although loss-of-function mutations, such as truncation and missense mutations, account for certain types of mutated KMT2D, KMT2D also appears to play an oncogenic role in a context- or tissue-specific manner [8, 9]. In in vitro cell line experiments, knockdown of KMT2D reduced the proliferation and invasion of pancreatic and breast cancer cells [12, 13]. In breast cancer cell line-derived xenografts, deficiency of KMT2D was correlated with improved prognosis in model animals [12]. More importantly, KMT2D not only catalyzes the methylation of H3K4 and acetylation of H3K27 but also mediates the activation of oncogenic enhancer and superenhancer signatures [14, 15]. Given the role of KMT2D in tumor promotion and suppression, however, the mechanisms by which KMT2D affects the development of patients with OSCC remain exceedingly unclear.

Dysregulation of the Wnt/β-catenin pathway represents a central signaling network that aggravates the stem-like properties of tumor cells [16–18]. In patient-derived organoids, a novel individual preclinical model that faithfully resembles the clinical features of parental tumors, the addition of Wnt signal-related agonists is essential for the establishment and long-term expansion of in vitro and in vivo organoid outgrowth [19–21]. As demonstrated by recent studies, oncogenic Wnt signal-related genes, particularly CTNNB1, are also mediated by epigenetic modulation [7]. For instance, a recent study revealed that the transcription of CTNNB1 in cancer cells was mediated by MEF2A, which can be occupied by KMT2D [14, 22]. Therefore, we aimed to determine whether KMT2D functions as a transcription coactivator of MEF2A to mediate stem-like properties in OSCC cells.

## Results

### Expression pattern of KMT2D in OSCC tumor specimens and cell lines

**Fig. 1:**
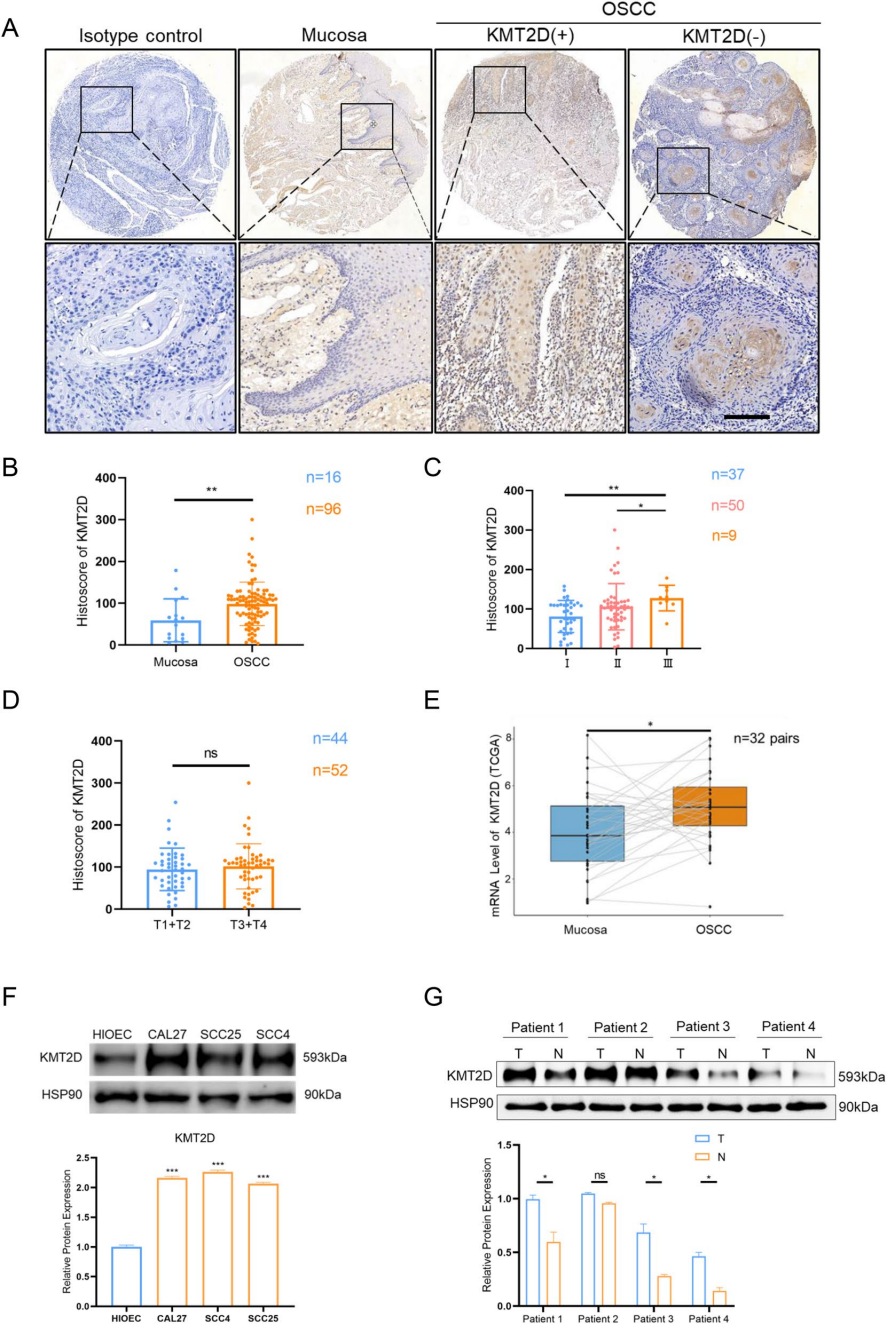
Expression pattern of KMT2D in patients with OSCC. **A** Representative immunohistochemical staining of KMT2D in normal mucosa and primary OSCC tissue of KMT2D(+) and KMT2D(−). (scale bars = 100 μm). **B** Quantification analysis of immunohistochemical histoscore of KMT2D among normal mucosa and OSCC. **C** KMT2D expression levels in OSCC tissues from different pathological grades. **D** KMT2D expression levels in OSCC tissues from different tumor sizes. **E** KMT2D mRNA expression levels of paired normal mucosa and OSCC from TCGA datasets. **F** KMT2D protein expression levels in different cell lines. **G** KMT2D protein expression in human OSCC tissues and adjacent normal mucosa from the same patient. Results are representative of at least three independent experiments (*p < 0.05, **p < 0.01). Data are presented as means ± SD. OSCC, oral squamous cell carcinoma; *SD* standard deviation,* ns* no significant,* KMT2D(+)* KMT2D positive,* KMT2D(−)* KMT2D negative

To profile the expression pattern of KMT2D in patients with OSCC, a panel of OSCC specimens (n = 96) and adjacent oral mucosa tissues (n = 16) were employed to conduct immunohistochemistry (IHC) staining for KMT2D (Fig. 1A). The results showed that the protein level of KMT2D was significantly elevated in OSCC specimens compared with oral mucosa tissues (Fig. 1B). To further confirm the role of KMT2D in the development of OSCC, we analyzed the staining intensity (i.e., histoscores) of KMT2D in OSCC specimens diagnosed with different tumor grades. The results demonstrated that the expression level of KMT2D gradually increased as the pathological grade of patients with OSCC increased (Fig. 1C, Additional file 1: Fig. S1A). However, there was no significant relationship between the expression level of KMT2D and tumor size (Fig. 1D). In line with these findings, analysis of the TCGA database confirmed that among 32 pairs of patient-derived samples, the expression of KMT2D at the mRNA level was stably upregulated in OSCC specimens compared with corresponding paracancerous tissues (Fig. 1E). Furthermore, the immunoblot results suggested that the expression of KMT2D in OSCC cell lines was significantly higher than that in HIOECs (Fig. 1F). Additionally, we extracted proteins from OSCC and normal tissues from four patients, and immunoblotting analysis showed that KMT2D expression was higher in OSCC than in normal tissue (Fig. 1G). Together, these results indicated that the expression of KMT2D may be correlated with the progression of OSCC.

### KMT2D sustains stem-like properties in patient-derived OSCC organoids

**Fig. 2:**
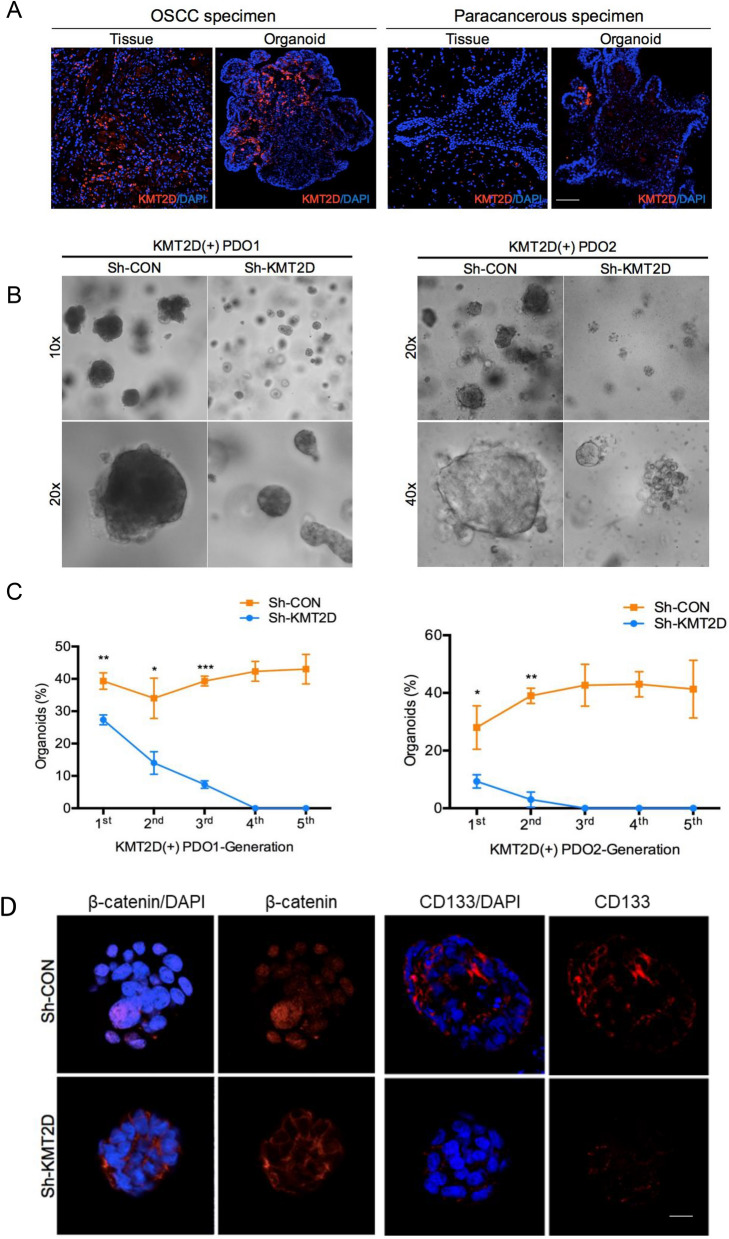
KMT2D contributes to the self-renewal ability of primary OSCC cells. **A** Representative images showed the immunofluorescence staining of KMT2D in patient-derived organoids generated from OSCC and paracancerous specimens, as well as their parental tissues. (scale bars = 50 μm). **B** Representative images indicated the organoid reconstitution assay (i.e. organoid morphology and clone size) conducted for OSCC cells transfected with Sh-CON and Sh-KMT2D. **C** Quantificational analysis showed the organoid reconstitution assay (i.e. organoid forming efficiency) conducted for OSCC cells transfected with Sh-KMT2D and Sh-CON. Student's t-test. **D** Representative images showed the immunofluorescence staining of β-catenin and CD133 in patient-derived OSCC organoids transfected with Sh-CON and Sh-KMT2D. (scale bars = 50 μm) Results are representative of at least three independent experiments(*p < 0.05,**p < 0.01, and ***p < 0.001). Data are presented as means ± SD. *OSCC* oral squamous cell carcinoma, *SD* standard deviation

To study the mechanisms of KMT2D in mediating the cellular biology of OSCC, several OSCC specimens (n = 6) were processed into single cells to generate patient-derived organoids (PDOs) according to our prevously described protocol [23]. Notably, the results of the immunofluorescence assay validated that the staining frequency and intensity of KMT2D were elevated in PDOs generated from OSCC compared to those from paracancerous specimens (Fig. 2A), indicating that PDOs resembled the expression pattern of KMT2D in parental tissues. To further investigate the function of KMT2D, primary OSCC cells were transfected with a short hairpin RNA (shRNA) targeting the expression of KMT2D (Sh-KMT2D). Decreased expression of KMT2D was confirmed by immunoblotting assays (Additional file 1: Fig. S1B). The results of the organoid reconstitution assay based on two PDO lines revealed that tumor cells transfected with sh-KMT2D formed fewer and smaller organoids during serial passage (Fig. 2B, C), suggesting that the expression of KMT2D contributes to the stem-like properties of primary OSCC cells. Furthermore, the results of the immunofluorescence assays in PDOs demonstrated that the expression of cancer stem cell markers, such as CD133 and β-catenin, was decreased when cells were transfected with Sh-KMT2D (Fig. 2D, Additional file 1: Fig. S1C).

### Expression of KMT2D aggravates malignant behaviors of OSCC cells

**Fig. 3:**
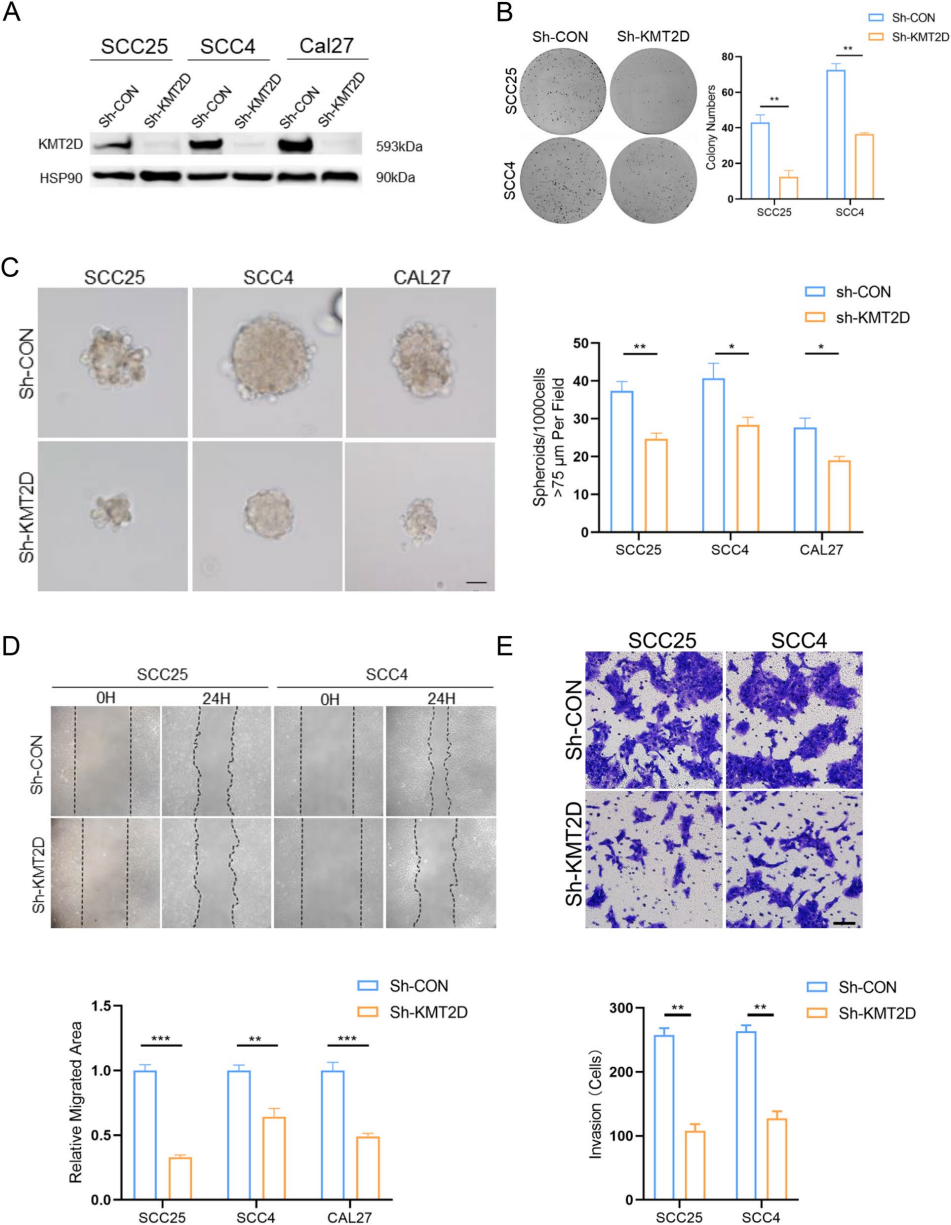
Expression of KMT2D aggravates malignant behaviors of OSCC cells. **A** Immunoblotting of KMT2D in SCC4, SCC25, and CAL27 cells respectively transfected with Sh-CON and Sh-KMT2D. **B** Colony formation assays and quantification analysis generated from SCC4 and SCC25 cells transfected with Sh-CON and Sh-KMT2D. **C** Sphere formation assays and quantification analysis carried out from SCC4, SCC25, and CAL27 cells transfected with Sh-CON and Sh-KMT2D. (scale bars = 50 μm). **D** Wound healing assays and quantification analysis generated from SCC4, SCC25, and CAL27 cells transfected with Sh-CON and Sh-KMT2D. (scale bars = 50 μm). **E** Invasion assays and quantification analysis generated from SCC4, SCC25 cells transfected with Sh-CON and Sh-KMT2D. (scale bars = 50 μm). Results are representative of at least three independent experiments (*p < 0.05, **p < 0.01 and ***p < 0.001). Data are presented as means ± SD; *SD* standard deviation

To more systematically understand the role of KMT2D in tumor biology, several OSCC cell lines, including SCC4, SCC25, and Cal27, were transfected with Sh-KMT2D. Decreased expression of KMT2D, H3K4me1 and H3K27ac in OSCC cell lines was confirmed, indicating the role of KMT2D as an epigenetic mediator in these models (Fig. 3A, Additional file 1: Fig. S1D). Then, multiple in vitro experiments, such as 2D clonal formation, 3D sphere formation, wound healing, and matrigel invasion assays, were carried out in OSCC cells transfected with Sh-KMT2D. As shown in Fig. 3B–E, knockdown of KMT2D in OSCC cells resulted in decreased colony formation, sphere formation, cell migration, and invasion efficiency compared to the corresponding cells that were transfected with the control vector (Sh-CON). In addition, immunoblotting assays of cancer stem cell markers, including CD133, OCT4, and ALDH1A1, showed a decrease as KMT2D was knocked down (Additional file 2: Fig. S2A). Taken together, these results demonstrate that the expression of KMT2D aggregates the malignant behaviors of OSCC cells in vitro.

### Knockdown of KMT2D impairs OSCC tumor growth in vivo

**Fig. 4:**
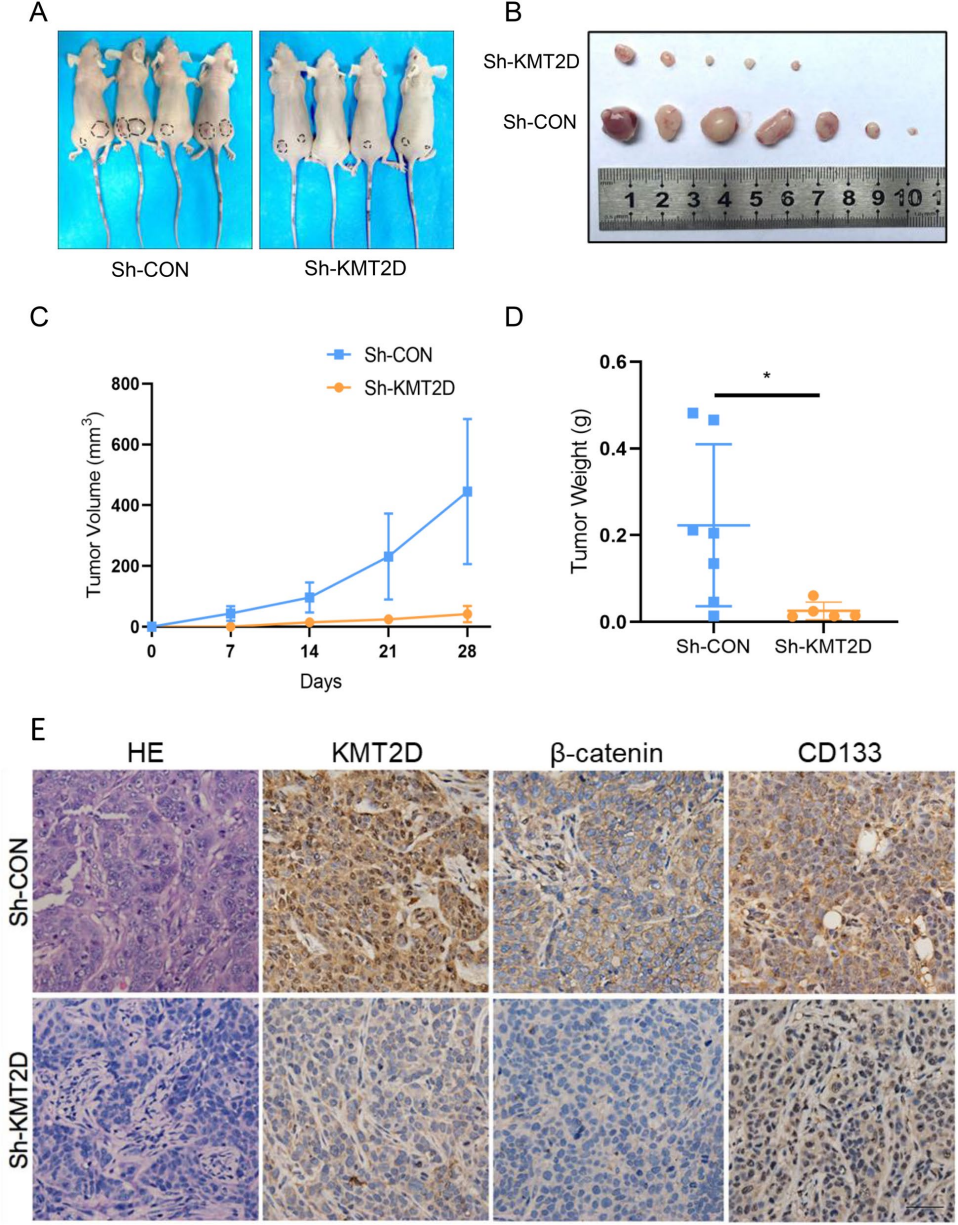
Knockdown of KMT2D impairs OSCC tumor growth in vivo*.* **A** Images of BALB/c nude mice from the two groups. **B** Images of xenograft tumors excised from BALB/c nude mice. **C** Quantification analysis of volumes of the xenograft tumors measured every 7 days on BALB/c nude mice. n = 8. **D** Weights of xenograft tumors excised from the BALB/c nude mice were evaluated. n = 8. **E** Representative hematoxylin and eosin (HE) and immunohistochemical staining of KMT2D and CSC-related markers in xenograft tumors. (scale bars = 50 μm). Results are representative of at least three independent experiments (*p < 0.05). *CS* cancer stem cell

To further investigate the role of KMT2D in OSCC progression, SCC4 cells transfected with Sh-KMT2D and control vector (Sh-CON) were employed to conduct in vivo transplantation assays. The results showed that knockdown of KMT2D gave rise to delayed growth dynamics of SCC4-derived xenografts, as confirmed by the decreased tumor volume and weight of xenografts initiated by SCC4 cells transfected with Sh-KMT2D, which were recorded at each observation time point (Fig. 4A–D). Furthermore, the results of the immunohistochemistry analysis verified that the expression of cancer stem cell markers, including CD133 and β-catenin, was decreased in SCC4-derived xenografts when KMT2D was knocked down (Fig. 4E). Collectively, these results demonstrated that the expression of KMT2D is essential for OSCC tumor growth in vivo.

### KMT2D cooperates with MEF2A to initiate CTNNB1 transcription

**Fig. 5:**
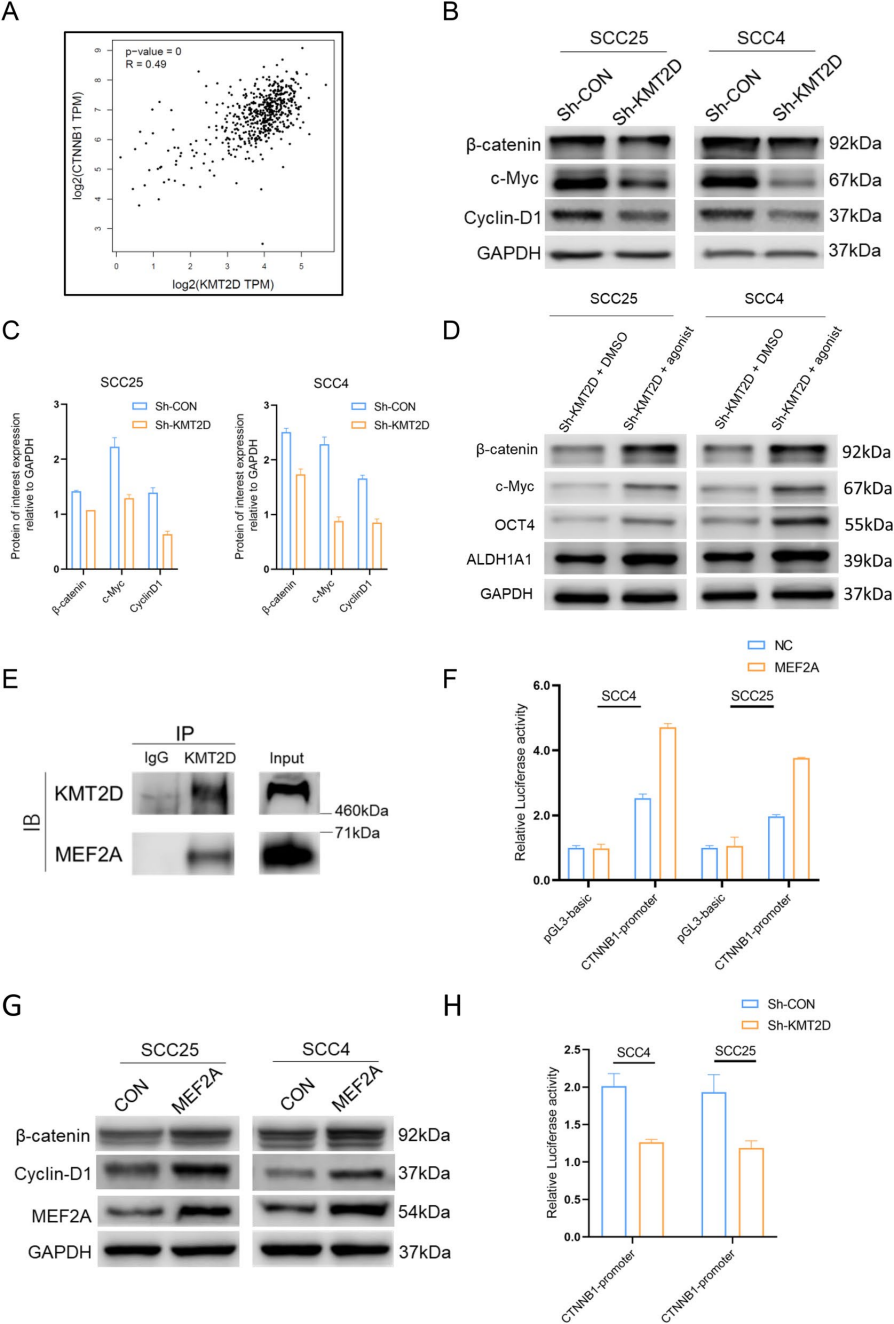
KMT2D cooperates with MEF2A to initiate the transcription of CTNNB1. **A** Correlation analysis of expression of KMT2D and β-catenin generated from GEPIA. **B** Immunoblotting and quantification analysis of β-catenin and downstream of canonical Wnt/β-catenin pathway in SCC4 and SCC25 cells transfected with Sh-CON and Sh-KMT2D. **C** Quantification analysis of immunoblotting of β-catenin and downstream of canonical Wnt/β-catenin pathway in SCC4 and SCC25 cells transfected with Sh-CON and Sh-KMT2D. **D** Immunoblotting of β-catenin and downstream of canonical Wnt/β-catenin pathway in SCC4 and SCC25 cells transfected with Sh-KMT2D treated with DMSO and Wnt/β-catenin agonist 1. **E** Co-IP experiments for KMT2D and MEF2A in SCC25 cells. **F** Relative transcriptional activity of CTNNB1 in SCC4 and SCC25 cells transfected MEF2A overexpression and control. **G** Immunoblotting of MEF2A, β-catenin, and CyclinD1 in SCC4 and SCC25 cells with MEF2A overexpression and control. **H** Relative transcriptional activity of CTNNB1 in SCC4 and SCC25 cells transfected with Sh-KMT2D and control. Data are presented as means ± SD. Results are representative of at least three independent experiments. *GEPIA* Gene Expression Profiling Interactive Analysis, *SD* standard deviation

Others and our previous studies have reported that sustaining Wnt/β-catenin activity plays a vital role in the maintenance of an aggressive phenotype in OSCC cells [18, 24]. Consistent with this, Gene Set Enrichment Analysis (GSEA) of the TCGA datasets revealed that the global expression of genes related to the Wnt/β-catenin signaling pathway was positively correlated with the level of KMT2D (Additional file 2: Fig. S2B). Specifically, the results of Gene Expression Profiling Interactive Analysis (GEPIA) further indicated that the expression of KMT2D was positively correlated with β-catenin, the key Wnt signaling-related genes. (Fig. 5A). To confirm the role of KMT2D in mediating Wnt/β-catenin in OSCC cells, several experiments were conducted in our preclinical models. The results of the immunoblotting assays demonstrated that the expression of β-catenin, CyclinD1, and c-Myc was reduced in OSCC cells (e.g., SCC4 and SCC25) transfected with Sh-KMT2D (Fig. 5B, C). To verify the role of the Wnt/β-catenin signaling pathway in KMT2D regulation of OSCC stemness, the Wnt/β-catenin agonist 1 was added to SCC4 and SCC25 cells transfected with Sh-KMT2D. The results of the immunoblotting assays revealed that Wnt/β-catenin agonist 1 could alleviate the promotive effects of KMT2D on β-catenin and downstream indicators of stem-like properties (Fig. 5D). β-Catenin is the key component of the Wnt/β-catenin signaling pathway [7]. Another study elucidated that MEF2A could directly upregulate CTNNB1 and enhance the activity of Wnt/β-catenin signaling in colorectal cancer [22]. Additionally, MEF2A has been revealed to function as a coregulatory transcription factor and be occupied by KMT2D, which binds to enhancers and promote transcription of downstream genes of MEF2A as a coactivator [14]. To elucidate the exact mechanism involved in the promotion of stem-like properties and activation of Wnt/β-catenin signaling by KMT2D in OSCC, co-immunoprecipitation (co-IP) was performed and demonstrated that when KMT2D was used as the IP target, MEF2A was pulled down (Fig. 5E), which confirmed that endogenous KMT2D could combine with MEF2A in OSCC cells. To verify whether MEF2A could directly regulate the transcriptional activity of CTNNB1, a dual-luciferase reporter assay was performed and showed significantly increased transcriptional activity in MEF2A-overexpressing cells (Fig. 5F), which indicated that MEF2A could regulate the transcription of CTNNB1 as a transcription factor. The results of the immunoblotting assay demonstrated that MEF2A overexpression upregulated the expression of β-catenin in SCC4 and SCC25 cells (Fig. 5G). Furthermore, to further evaluate the interaction between KMT2D and MEF2A and the effect of KMT2D on CTNNB1 transcriptional activity, a dual-luciferase reporter assay was performed in OSCC cells transfected with Sh-KMT2D. The result showed that transcription activity of CTNNB1 was downregulated when KMT2D was knockdown (Fig. 5H). Taken together, these data suggest that KMT2D acts coordinately with MEF2A in regulating CTNNB1 transcription.

## Discussion

In this study, OSCC tissue microarrays, patient-derived organoid platforms, and in vitro and in vivo cancer cell line experiments were integrated to systematically understand the biological scenario of KMT2D in tumor development. As a result, we found that the upregulation of KMT2D not only correlated with a more advanced pathologic tumor grade but also contributed to aggressive cancer cell behaviors, providing a novel rationale for targeting KMT2D function in patients with OSCC.

KMT2D is involved in the KMT2 family proteins that methylate lysine 4 on the histone H3 tail (H3K4) at important regulatory regions of the genome in eukaryotes, which contribute largely to the cellular transcription network by modulating chromatin structures and DNA accessibility [8]. As the largest H3K4 methyltransferase among them, KMT2D also represents the most frequently mutated gene in multiple human cancers, including OSCC [1, 2, 8]. Nonetheless, the role of KMT2D in tumor development remains largely controversial [8, 9]. Using a gene-editing mouse model, Dhar and Alam showed that tissue-specific ablation of *Kmt2d* in the brain and lung promoted tumorigenesis [25, 26]. On the other hand, Kim and Abudureheman revealed the tumor-promoting effects of KMT2D in cell lines and xenograft models [10, 27]. Here, we found that the expression of KMT2D was elevated in OSCC specimens compared to adjacent tissues and, more importantly, was associated with increased tumor grade. Using a patient-derived organoid model, which strongly resembled the expression pattern of KMT2D in parental OSCC specimens, we observed that knockdown of KMT2D impaired the self-renewal of primary OSCC cells. Further experiments on several OSCC cell lines, in which KMT2D functions as a regulator of H3K4me1 and H3K27ac levels, also confirmed that the expression of KMT2D contributed to the stem-like properties, metastatic potential, and in vivo tumor growth ability of OSCC cells. In conclusion, our data clearly illustrated the protumor effects of KMT2D in OSCC.

The WNT signal transduction landscape, particularly canonical Wnt/β-catenin signaling, is a master regulator of individual cellular fate throughout human life [1]. In patients with cancer, extensive studies have reported that the dysregulation of Wnt/β-catenin signaling is not only a passive result of malignant mutations but also a mighty trigger of an aggressive phenotype in cancer cells [17, 18, 24]. In support of this, investigations by Xie and colleagues confirmed that SOX8-mediated Wnt/β-catenin pathways gave rise to stem-like properties, cisplatin resistance and an epithelial-to-mesenchymal transition phenotype in tongue squamous cell carcinoma [18]. In a recent study by Xiao et al., MEF2A, as a member of the MEF2 family of transcription factors, was shown to directly bind to the promoter region to initiate the transcription of CTNNB1 and induce the activation of WNT/β-catenin signaling in colorectal cancer [22]. In addition, Liu and his colleagues confirmed that KMT2D functions as a coactivator of MEF2A to activate the transcription of *myh7* in mice [14]. Here, we provide evidence, based on bioinformatics analysis and experimental validation, that KMT2D can bind to MEF2A to inhibit the transcription of CTNNB1 and enhance the activation of Wnt/β-catenin signaling in OSCC.

Our study has limitations. Considering the presence of KMT2D-mutated/deficient tumors [25], the role of mutated KMT2D in mediating Wnt/β-catenin pathways, as well as the role of KMT2D as a mediator of oncogenic enhancer and superenhancer signatures in OSCC, were not discussed in the current study and deserve further investigation [28–30]. Additionally, KMT2D has widespread functions as a histone methyltransferase, and other mechanisms that regulate the self-renewal of oral cancer stem cells and promote the progression of OSCC are imperative for further illuminated. However, we reported for the first time that in the patient-derived organoid platform, which was supposed to advance to precision medicine, the upregulation of KMT2D contributes to the stem-like properties of OSCC cells by sustaining Wnt/β-catenin activity. Overall, we provided a new window to more comprehensively determine the therapeutic potential of KMT2D function in patients with OSCC.

## Conclusions

In this study, we found that upregulation of KMT2D aggravates malignant behaviors in a patient-derived organoid (PDO) platform and in in vitro and in vivo cancer cell lines by mediating the transcription of CTNNB1 in cooperation with MEF2A thus sustaining stem-like properties and Wnt/β-catenin signaling. Our studies provide a new molecular insight into the epigenetic regulation of the Wnt/β-catenin pathway and malignant behaviors of OSCC.

## Materials and methods

### Cell lines and cultures

The OSCC cell lines CAL27, SCC25 and SCC4 were obtained from the China Center for Type Culture Collection (Shanghai, China). CAL27 was maintained in Dulbecco’s modified Eagle’s medium (DMEM)/High Glucose. SCC25 and SCC4 were cultivated in DMEM:F12 (1:1). Ten percent fetal bovine serum (FBS) was added to the medium for all cell lines.

### Human tissue samples and tissue microarrays

The study was approved by the Ethics Committee of School and Hospital of Stomatology, Wuhan University. Written informed consent was obtained from all patients. The chips used in this study included samples of 96 human OSCC tissues and 16 normal oral mucosa obtained from the School and Hospital of Stomatology, Wuhan University. None of the patients received preoperative treatment.

### Collection of primary OSCC cells

Fresh specimens were broken into small sections with scissors. Small sections were all cultivated in serum‑free DMEM:F12 (1:1) containing 1.5 mg/ml collagenase IV (Gibco; Thermo Fisher Scientific.), 20 μg/ml hyaluronidase (Sigma‑Aldrich.), and 1% penicillin/streptomycin (Thermo Fisher Scientific) at 37 °C for 1 to 2 h. To exclude red blood cells, red blood cell lysis buffer was used for cells on ice for 10 min. Next, PBS was used to wash the cells twice. Single primary OSCC cells were applied in subsequent organoid cultures.

### Lentiviral transfection

Cal-27, SCC-25, and SCC4 cells were transfected with KMT2D short hairpin RNA (shRNA) named Sh-KMT2D, and a scramble shRNA control vector was also used named Sh-CON. SCC-25 and SCC4 cells were transfected with recombinant lentiviruses to stably overexpress MEF2A. The lentivirus was purchased from GeneChem (Shanghai, China). Forty-eight hours after incubation with lentivirus, the cells could be observed for fluorescence. To identify stable KMT2D knockdown and MEF2A overexpression cell lines, the transfected cells were cultured with 2 μg/ml puromycin for 7 days. KMT2D knockdown expression and MEF2A overexpression were then evaluated with western blotting.

### Organoid culture

Primary OSCC cells diluted to 5000 cells/30 µl were embedded in Matrigel (BD Biosciences) in 24-well plates. Organoid cultures were performed as previously described [23, 24]. Organoids > 50 μm were counted. Forming efficiency (%) = scored organoid number/total plated OSCC cells.

### Sphere formation assay

One thousand cells were seeded in each well of 12-well plates pretreated with a poly HEMA solution (10 g/l in 95% ethanol; Millipore Sigma). Cells were cultivated in cancer stem cell medium: DMEM-F12 supplemented with 2% B27 supplement (Life Technologies, Waltham, MA, USA), 100 ng/ml streptomycin, 100 U/ml penicillin, 10 ng/ml human bFGF (PeproTech), and 20 ng/ml human EGF (PeproTech). Ten days later, the cells were photographed, and tumorspheres > 75 μm were counted.

### Colony formation assay

One thousand cells were seeded in 6-well plates and cultivated in medium supplemented with 10% FBS. Ten days later, the cells were washed twice with PBS, 4% methanol was used to immobilize the cells, and crystal violet was applied to stain the colonies. Six-well plates were photographed under a light microscope after drying. The colonies consisting of more than 50 cells were counted.

### Matrigel invasion assays

Transwell chambers with a polycarbonate membrane with 8 μm pores (Corning, USA) were coated with Matrigel basement membrane matrix (Corning, 356,237, USA). Then, 600 µl medium containing 10% FBS was placed into the lower chambers, and 2 × 105 cells suspended in FBS-free medium were seeded into the upper chambers. Forty-eight hours later, the medium was discarded, and the cells invading the lower chamber were stained and counted.

### Antibodies, immunohistochemistry, and evaluation

Immunohistochemistry studies were conducted using the following antibodies. Anti-KMT2D antibody (dilution 1:100, #27,266, Proteintech); anti-β-catenin antibody (dilution 1:2000, #51,067, Proteintech); anti-CD133 antibody (dilution 1:1000, #18,470, Proteintech); anti-c-Myc antibody (dilution 1:100, ab32072, Abcam). Histologic sections on human tissue microarrays were cut at 5 μm. The immunochemical test kit was from MaiXin Ltd. (Fu Zhou, China). Peroxidase blocker and 5% goat serum were added in sequence to histologic sections, after which the primary antibody was incubated overnight at 4 °C. The corresponding secondary antibody and avidin–biotin-peroxidase were then added, and DAB and hematoxylin were employed for visualization. We used Aperio ImageScope software (Version 9.1; Leica, Wetzlar, Germany) for analysis.

### Immunofluorescence assay and western blot analysis

Immunofluorescence and immunoblotting were performed as previously described [19]. The following antibodies were used for immunoblotting. Anti-KMT2D antibody (dilution 1:1000, #ABE1867, Sigma); anti-CD133 antibody (dilution 1:1000, #18,470, Proteintech); anti-ALDH1A1 antibody (dilution 1:2000, #15,910, Proteintech); anti-OCT4 antibody (dilution 1:1000, #11,263, Proteintech); anti-β-catenin antibody (dilution 1:2000, #51,067, Proteintech); anti-c-Myc antibody (dilution 1:100, #ab32072, Abcam); anti-Cyclin D1 antibody (dilution 1:1000, #26,939, Proteintech); anti-H3K4me1 antibody (dilution 1:500, #ab8895, Abcam); and anti-H3K27ac antibody (dilution 1:500, #ab4729, Abcam); anti-MEF2A antibody (dilution 1:500, #12,382, Proteintech). The following antibodies were used for immunofluorescence. Anti-KMT2D: antibody (dilution 1:100, #27,266, Proteintech); anti-CD133 antibody (dilution 1:200, #18,470, Proteintech); anti-β-catenin antibody (dilution 1:200, #51,067, Proteintech).

### Animal experiments

All animal studies were approved by the Ethics Committee of Wuhan University. Eight BALB/c nude mice (4–6 weeks, 18–20 g) purchased from Beijing Vital River Laboratory Animal Technology Co., Ltd. (Beijing, China) were separated into two groups (n = 4). SCC4 cells transfected with Sh-KMT2D and ShCON mixed in 50 µl PBS and 50 µl Matrigel were injected subcutaneously into both the left and right back of each mouse (5 × 10^5^ cells per tumor). Tumor volumes were measured and calculated every 7 days using the formula (width2 × length)/2. After 28 days, the mice were euthanized, and the tumor load was removed and further analyzed.

### Luciferase reporter assay

The pGL3-basic plasmid containing the CTNNB1 promoter, the pGL3-basic luciferase plasmid, and the Renilla luciferase plasmid were purchased from Miaolingbio (Wuhan, China). The pGL3-Basic plasmid inserted with the promoter sequence of CTNNB1 and pGL3-Basic plasmid were cotransfected with Renilla luciferase plasmid into SCC4 and SCC25. Forty-eight hours later, luciferase activity was measured by using a Dual-Luciferase Reporter Assay Kit (Promega, Madison, MI, USA).

### Co-immunoprecipitation

Universal Magnetic co-IP kits were purchased from Active Motif (54002, USA). Co-immunoprecipitation was performed following the manufacturer’s instructions. Five micrograms of anti-KMT2D antibody and anti-IgG antibody were added to each sample for protein pull-down. Immunoblotting was performed as previously described.

### Statistical analysis

All the results were given in triplicate and repeated at least 3 times. All analyses were carried out using Student's t-tests by SPSS software (SPSS V.17.0, Chicago, Ill, USA) and GraphPad Prism software 8.3.0 (GraphPadSoftware, Inc., San Diego, CA, USA). Statistical significance was defined as p < 0.05.

## Supplementary Information

**Additional file 1: Figure S1**. **A** Representative immunohistochemical staining of KMT2D in primary OSCC tissues of various histological grades. **B** Immunoblotting of KMT2D in the primary OSCC cells transfected with Sh-CON and Sh-KMT2D.** C** Quantification analysis of immunofluorescence staining of β-catenin and CD133 in patient-derived OSCC organoids transfected with Sh-CON and Sh-KMT2D. **D** Immunoblotting and quantification analysis of H3K4me1 and H3K27ac in SCC4 and SCC25 cells transfected with Sh-CON and Sh-KMT2D. Results are representative of at least three independent experiments.

**Additional file 2: Figure S2**. **A** Immunoblotting and quantification analysis of cancer stem cells markers including CD133, OCT4, ALDH1A1 in SCC4, SCC25, and CAL27 cells transfected with Sh-CON and Sh-KMT2D. **B** GSEA enrichment analysis of KMT2D on the TCGA datasets. Results are representative of at least three independent experiments. GSEA, Gene Set Enrichment Analysis; TCGA, The Cancer Genome Atlas.

## Data Availability

The datasets used and analysed during the current study are available from the corresponding author on reasonable request.

## Abbreviations

OSCC: Oral squamous cell carcinoma
HMT: Histone methyltransferase
KDM: Histone lysine demethylases
KMT2D: Histone-lysine *N*-methyltransferase 2D
DKK1: Dickkopf-related protein 1
IHC: Immunohistochemistry
TCGA: The cancer genome atlas
PDO: Patient-derived organoids
Sh-RNA: Short hairpin RNA
GSEA: Gene set enrichment analysis
GEPIA: Gene expression profiling interactive analysis
Co-IP: Co-immunoprecipitation
