# Supplementary figures and images for: Histone methyltransferase KMT2D cooperates with MEF2A to promote the stem-like properties of oral squamous cell carcinoma
Source: Cell Biosci. 2022 Apr 27;12:49. doi: 10.1186/s13578-022-00785-8 (PMC9044881; doi:10.1186/s13578-022-00785-8)

A

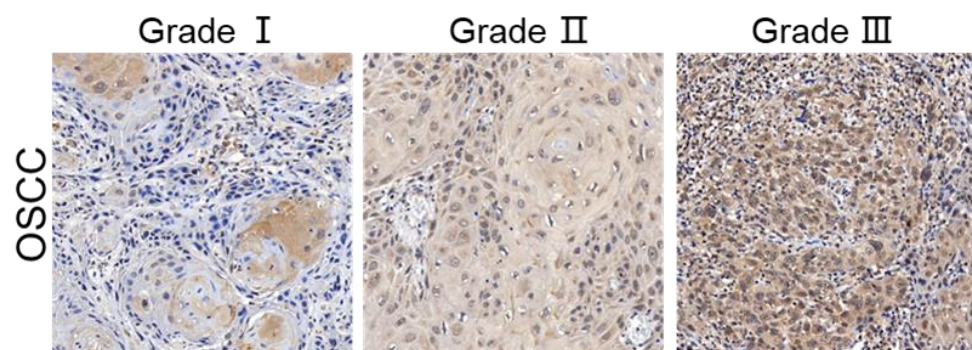

B

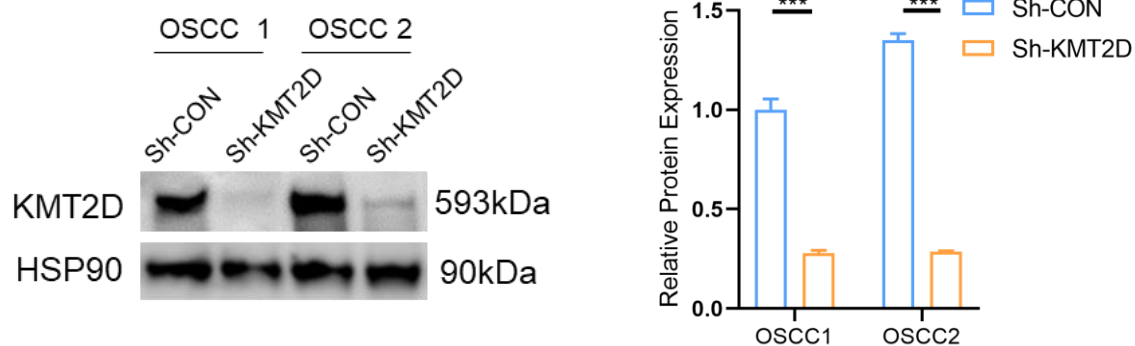

C

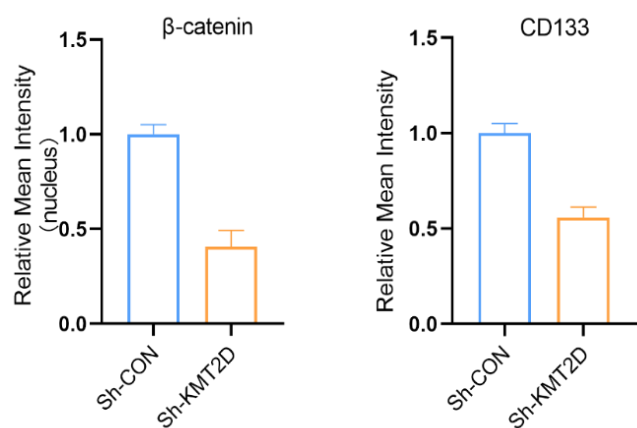

D

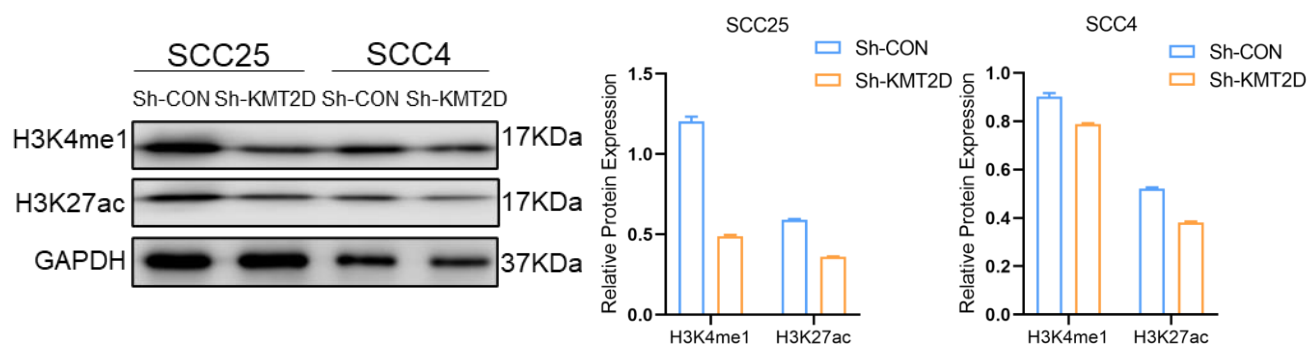

Supplement: Supplementary file 1 — Additional file 1: Figure S1. A Representative immunohistochemical staining of KMT2D in primary OSCC tissues of various histological grades. B Immunoblotting of KMT2D in the primary OSCC cells transfected with Sh-CON and Sh-KMT2D. C Quantification analysis of immunofluorescence staining of β-catenin and CD133 in patient-derived OSCC organoids transfected with Sh-CON and Sh-KMT2D. D Immunoblotting and quantification analysis of H3K4me1 and H3K27ac in SCC4 and SCC25 cells transfected with Sh-CON and Sh-KMT2D. Results are representative of at least three independent experiments. [file 13578_2022_785_MOESM1_ESM.pdf]

## Slide 1
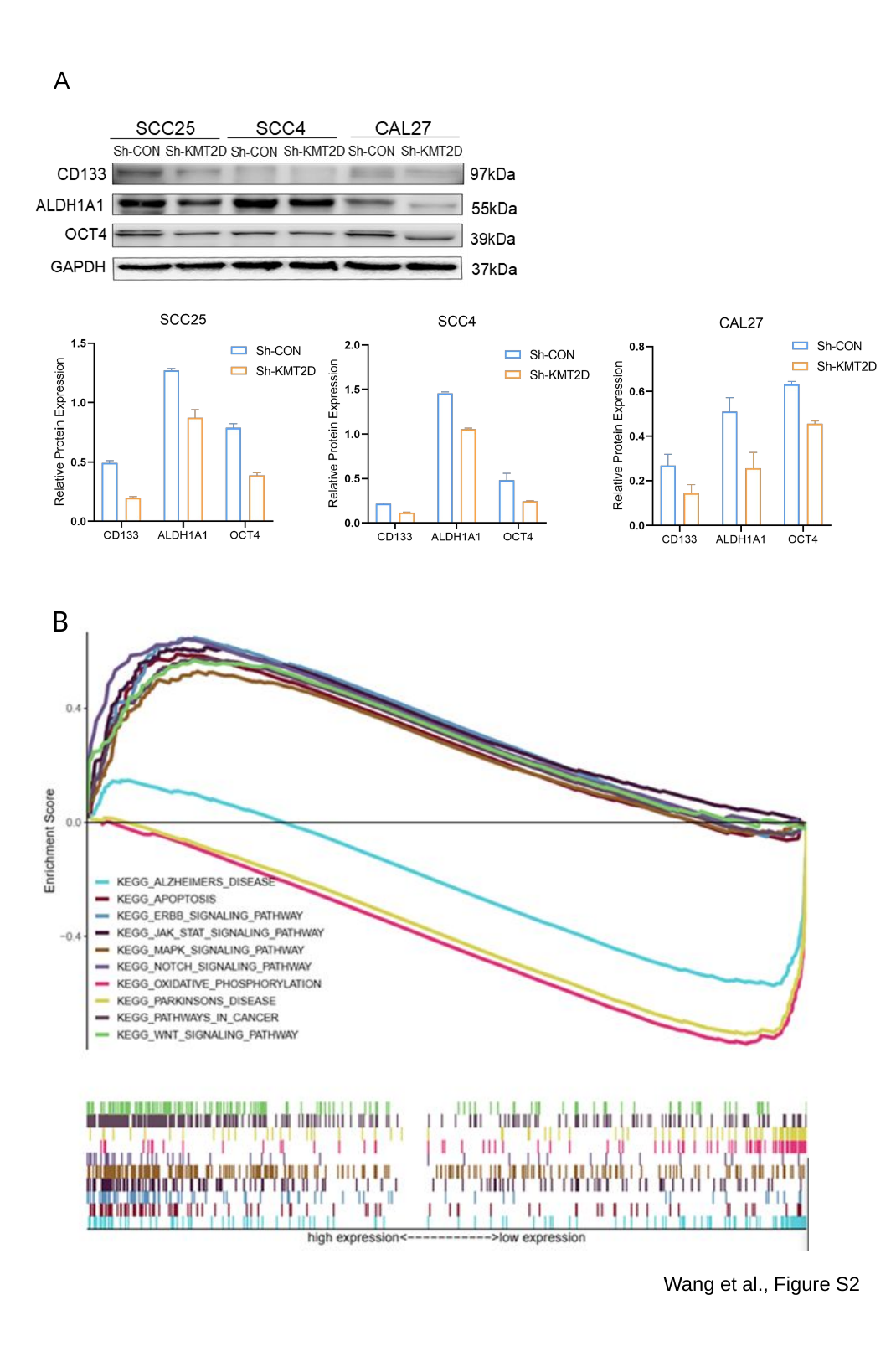

A
B
Wang et al., Figure S2

Supplement: Supplementary file 2 — Additional file 2: Figure S2. A Immunoblotting and quantification analysis of cancer stem cells markers including CD133, OCT4, ALDH1A1 in SCC4, SCC25, and CAL27 cells transfected with Sh-CON and Sh-KMT2D. B GSEA enrichment analysis of KMT2D on the TCGA datasets. Results are representative of at least three independent experiments. GSEA, Gene Set Enrichment Analysis; TCGA, The Cancer Genome Atlas. [file 13578_2022_785_MOESM2_ESM.pptx]
